# Supplementary material for: Radiomics for the Prediction of Response to Antifibrotic Treatment in Patients with Idiopathic Pulmonary Fibrosis: A Pilot Study
Source: Diagnostics (Basel). 2022 Apr 15;12(4):1002. doi: 10.3390/diagnostics12041002 (PMC9028756; doi:10.3390/diagnostics12041002)
Supplement: Supplementary file 1 [file diagnostics-12-01002-s001.zip › diagnostics-1665595-supplementary.pdf]

Table S1. Acquisition Protocols and Technical Parameters for HRCT Scans in the Two Institutions.

	**Institution 1**	**Institution 2**
Multi-detector CT scanners	256-slice Somatom Definition Flash (Siemens Healthcare, Forchheim, Germany), 128-slice Somatom Definition AS (Siemens Healthcare)	256-slice Somatom Definition Flash (Siemens Healthcare, Forchheim, Germany), 128-slice Somatom Definition AS (Siemens Healthcare), 16-slice Somatom Emotion (Siemens Healthcare), 256-slice Brilliance iCT (Philips Healthcare, Cleveland, USA)
Tube voltage (kVp)	120	120
Gantry rotation speed (s/r)	0.5	0.5 or 0.6
Collimation (mm)	128 × 0.6 or 256 × 0.6	16 × 0.6 or 128 × 0.6 or 256 × 0.6
Reconstructed slice thickness (mm)	1.5	1.5
Reconstruction kernel	sharp	sharp
